# Physical activity and short-form video addiction among adolescents: the chain mediating role of cognitive flexibility and anxiety

**DOI:** 10.3389/fpubh.2026.1854722

**Published:** 2026-08-14

**Authors:** Mofei Wen, Ning Wang, Hongqing Xiao, Yang Liu, Xiaomei Niu

**Affiliations:** 1 College of Physical Education, Chengdu University, Chengdu, China; 2 Chengdu Vocational University of the Arts, Chengdu, China; 3 Jishou University, Jishou, China; 4 Chengdu Experimental Middle School, Chengdu, China; 5 The Department of Pharmacy, The Second People's Hospital of Longgang District, Shenzhen, China

**Keywords:** adolescents, anxiety, cognitive flexibility, physical activity, short-form video addiction

## Abstract

**Background:**

Short-form video platforms have become a common source of entertainment and social interaction among adolescents. Excessive or poorly controlled use may be associated with adverse psychological and behavioral outcomes. This study examined the association between physical activity and short-form video addiction and tested whether cognitive flexibility and anxiety statistically mediated this association.

**Methods:**

A cross-sectional study was conducted during the autumn term of 2023 via a convenience sampling method from three secondary institutions located in Hunan Province, China. The resultant sample comprised 492 adolescents (236 male, 256 female), with an average age of 15.36 years (SD = 0.54). Self-reported measures of physical activity, cognitive flexibility, anxiety, and short-form video addiction were collected and analyzed using a mediation model.

**Results:**

Physical activity was negatively correlated with short-form video addiction (*r* = −0.274, *p* < 0.001). Chain mediation analysis showed a significant serial indirect association linking physical activity with short-form video addiction through cognitive flexibility and anxiety (effect size = −0.029, SE = 0.009, 95% CI [−0.050, −0.013]), accounting for 13.94% of the total association. Cognitive flexibility (effect size = −0.049, 95% CI [−0.088, −0.019]) and anxiety (effect size = −0.041, 95% CI [−0.079, −0.010]) also showed significant single-mediator indirect associations. The total indirect association accounted for 57.69% of the overall association.

**Conclusions:**

Higher physical activity was associated with lower short-form video addiction among the adolescents studied, and this association was partly explained by cognitive flexibility and anxiety. Because the study was cross-sectional, the findings should be interpreted as correlational evidence rather than proof of temporal or causal mechanisms.

## 1 Background

Amid the rapid proliferation of short-form video platforms, such as TikTok, Instagram Reels, and YouTube Shorts (1), short-form video content has become a common channel for social interaction and leisure among adolescents. Short-form video addiction refers to excessive and difficult-to-control dependence on short-form video platforms accompanied by psychological craving (2). The immediacy, personalized recommendation algorithms, and highly stimulating content streams of these platforms may reinforce repeated use, particularly among adolescents whose self-regulatory capacities are still developing (3, 4). Recent national reports indicate that short-form video users account for a very large proportion of internet users in China (5), and empirical studies have reported substantial screening rates of problematic short-form video use among Chinese adolescents (6, 7). Symptoms may include impaired attentional control, craving, withdrawal-like reactions, and emotional or social problems when use becomes excessive (8). This phenomenon may be further intensified by algorithmic recommendation systems that repeatedly deliver personally attractive content (9). Given the large adolescent user base and the developmental vulnerability of this age group, identifying protective factors against short-form video addiction is an important public-health and educational issue.

Physical activity is widely recognized as a health-related behavior associated with adolescents' physical, cognitive, and emotional development (10). It refers to any bodily movement produced by skeletal muscles that requires energy expenditure (11). In research on problematic digital media use, adolescents who report more regular physical activity often show stronger self-regulation, better affective adjustment, and lower levels of addictive-behavior symptoms (12, 13). These findings suggest that physical activity may be meaningfully related to short-form video addiction, although the direction of this relationship cannot be established from cross-sectional evidence (14). Several non-causal explanations are plausible (15). First, adolescents who spend more time in physical activity may have more structured offline routines and less unstructured leisure time available for repeated short-form video viewing. Second, physical activity may co-occur with experiences of competence, mastery, peer interaction, and positive affect, which may make algorithm-driven digital rewards less dominant in daily life (16). Third, higher physical activity may be a marker of broader healthy routines, including better sleep, school engagement, and family supervision, which were not fully measured in the present study (17). Based on this literature, the present study hypothesized that physical activity would be negatively associated with short-form video addiction.

Cognitive flexibility may be one cognitive factor linking physical activity with short-form video addiction. Cognitive flexibility refers to the capacity to adjust cognitive strategies and behavioral responses when environmental demands change (18). Prior studies have reported that screen-based sedentary behavior is negatively associated with cognitive flexibility (19), whereas physical activity is positively associated with adolescents' cognitive flexibility (20, 21). Strategic or organized physical activity may also be associated with more flexible attention shifting and goal-directed behavior among young people with problematic Internet-related behaviors (22). From an interpretative perspective, adolescents with higher cognitive flexibility may be better able to shift attention away from salient short-form video cues, reconsider immediate viewing impulses, and align behavior with academic or life goals (23–25). Conversely, lower cognitive flexibility may be associated with greater difficulty disengaging from highly stimulating digital content (24, 25). Therefore, this study hypothesized that cognitive flexibility statistically mediates the association between physical activity and short-form video addiction.

Anxiety is a negative emotional state that is unsettling, tense, apprehensive, fearful, or worried, often accompanied by physiological reactions such as a racing heart and tight muscles (26). In today's society, individuals are faced with a variety of stressors, including work pressure, social anxiety and uncertainty in life, which together lead to the prevalence of anxiety (27). The escape theory points out that individuals often escape from realistic pressure through cognitive deconstruction, so as to avoid negative experiences (28). The motivation of escape mechanism explains the psychological causes of individuals' excessive use of the Internet and short-form videos in the face of anxiety (29). The relaxation and entertainment of short-form videos can provide individuals with immediate pleasure, so that they can temporarily forget the negative emotions they have encountered. However, this behavior also heightens the propensity for short-form video addiction, which thereby may affect an individual's daily life and interpersonal relationships (30). On the contrary, physical activity functions crucially in the alleviation of negative emotions such as anxiety by helping to (31) release endorphins (32) and other neurotransmitters, which boost mental health and also improve overall wellbeing. By engaging in physical activity, individuals can somewhat reduce the need for short-form videos as a form of escape. This positive cycle not only helps to alleviate anxiety, but also prevents short-form video addiction from occurring (33). Given the aforementioned evidence, the current study hypothesized that anxiety statistically mediates the association between physical activity and short-form video addiction.

Cognitive flexibility and anxiety are closely interrelated. Adolescents with lower cognitive flexibility may have more difficulty shifting perspectives, solving problems flexibly, and adapting to stressful situations, which may correspond to higher anxiety (34, 35). At the same time, anxiety may narrow attentional focus and make flexible thinking more difficult, indicating that the association may be reciprocal rather than unidirectional (36). In the present model, cognitive flexibility was placed before anxiety because it represents an executive and coping-related capacity that may be associated with how adolescents appraise and manage stress. Nevertheless, given the cross-sectional design, this order should be understood as theoretically specified rather than empirically confirmed (37). Longitudinal studies are needed to compare whether cognitive flexibility precedes anxiety, anxiety precedes cognitive flexibility, or both processes influence each other over time.

At the same time, anxiety may narrow attentional focus and make flexible thinking more difficult, indicating that the association may be reciprocal rather than unidirectional. In the present model, cognitive flexibility was placed before anxiety because it represents an executive and coping-related capacity that may be associated with how adolescents appraise and manage stress. Nevertheless, given the cross-sectional design, this order should be understood as theoretically specified rather than empirically confirmed. Longitudinal studies are needed to compare whether cognitive flexibility precedes anxiety, anxiety precedes cognitive flexibility, or both processes influence each other over time.

**Figure 1:**
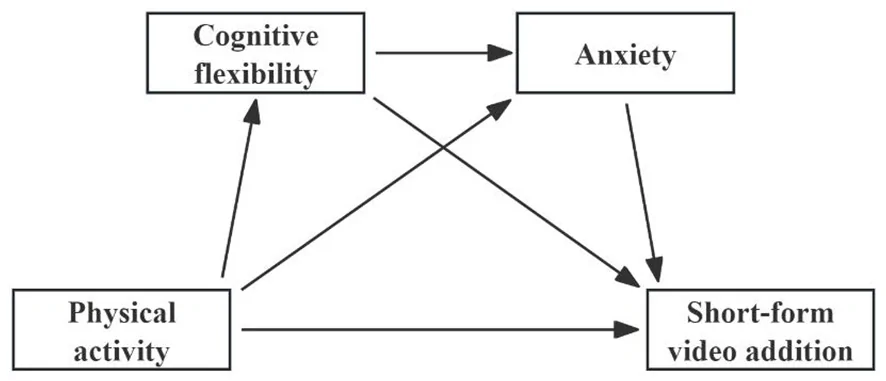
Hypothetical chain mediation model.

The study tested the following hypotheses: H1, physical activity is negatively associated with short-form video addiction among adolescents; H2, cognitive flexibility statistically mediates the association between physical activity and short-form video addiction; H3, anxiety statistically mediates the association between physical activity and short-form video addiction; and H4, cognitive flexibility and anxiety form a serial indirect pathway linking physical activity with short-form video addiction. Therefore, we propose a hypothetical chain mediation model (Figure 1).

## 2 Methods

### 2.1 Participants

This cross-sectional study used convenience sampling to collect data during the autumn term of 2025 from three secondary schools in Hunan Province, China. A total of 571 adolescents were initially enrolled. Following the sampling principle proposed by Kline, the study required at least 10 respondents per questionnaire item (38). With 35 scale items or calculated indicators included in the main measures (physical activity: three calculated indicators; short-form video addiction: 13 items; anxiety: seven items; cognitive flexibility: eight items; plus demographic variables), the minimum recommended sample size was 350. The final valid sample of 492 participants exceeded this threshold and was considered adequate for the planned mediation analysis, although the single-province convenience sample limits external generalizability. The study was approved by the Biomedical Ethics Committee of Jishou University. Participation was voluntary and anonymous, and no incentives were provided. Before questionnaire distribution, trained research assistants explained the purpose of the study, confidentiality procedures, expected completion time, and the right to withdraw at any point. Written assent from adolescents and informed consent from their legal guardians were obtained. Data screening excluded 79 questionnaires from the 571 initially distributed questionnaires. Exclusion criteria were: (1) incomplete responses, with listwise deletion applied to questionnaires containing any unanswered items; and (2) evident low-quality response patterns, including selecting the same option for two or more consecutive scales or showing a wave-like pattern across the questionnaire. Because detailed records of exclusions by specific criterion were not retained during the original screening process, only the total number of excluded questionnaires could be reported. This limitation has been explicitly acknowledged in the Limitations section of the revised manuscript. The final valid sample included 492 adolescents (236 males and 256 females; 78 only children and 414 non-only children), with a mean age of 15.36 years (SD = 0.54). Gender, age, and only-child status were included as covariates in the mediation model. Additional school-level characteristics, including urban/rural location, public/private status, and socioeconomic composition, were not available in the original survey. Therefore, the single-province convenience sample should be considered when interpreting the external validity of the findings.

### 2.2 Measures

#### 2.2.1 Physical activity

Physical activity was measured using the Physical Activity Scale originally developed by Hashimoto (39) and revised by Liang (40). The scale has been widely used in Chinese physical-activity research, including studies of adolescents and young people examining physical activity in relation to digital media use, internet addiction, anxiety, and related psychological outcomes (41, 42). The scale assesses three indicators: intensity, duration, and frequency (e.g., “How often do you engage in the above-mentioned physical activity?”). Intensity and frequency are scored from one to five, whereas duration is scored from one to four. The total physical activity score is calculated as intensity x duration x frequency; higher scores indicate a higher level of physical activity. In the present sample, Cronbach's alpha for the three calculated indicators was 0.67. Although this value is below the conventional 0.70 criterion, methodological literature notes that Cronbach's alpha is sensitive to the number of items and that, when a construct is measured with a small number of indicators (e.g., fewer than six items), an alpha above 0.60 may still be considered acceptable for exploratory or applied research (43). Therefore, the internal consistency of the Physical Activity Scale in this study was considered marginally acceptable, and findings involving physical activity were interpreted with caution. Because the total physical activity score was calculated as the product of intensity, duration, and frequency, its distribution was examined prior to analysis. The physical activity score showed a skewness of 1.471 and a kurtosis of 1.240, both of which fell within commonly accepted ranges and did not indicate severe non-normality. Therefore, no transformation of the physical activity score was applied. Furthermore, the mediation analysis was conducted using the PROCESS macro with 5,000 bootstrap resamples, which does not require the indirect effect to follow a normal distribution. Accordingly, the original validated scoring method was retained. Although the internal consistency was considered marginally acceptable for a three-indicator scale, measurement error may have attenuated the observed associations involving physical activity; thus, these estimates should be interpreted cautiously.

#### 2.2.2 Short-form video addiction

In order to assess the degree of short-form video addiction among the participants, the Problematic Short-form Video Media Use Scale—authored by Mao and Jiang (44), which encompasses three dimensions: knowledge and behavioral changes, physiological discomfort, and social viscosity (e.g., “Once I open a short-form video app, I find it hard to close it immediately—I always end up wanting to watch just a bit more before I feel satisfied.”). This scale encompasses 13 items graded on a 5-point Likert scale (1 = “very unsuitable”, 5 = “very suitable”). The overall item score functioned as the measure of short-form video addiction, with elevated scores reflecting greater severity. For the present sample, Cronbach's alpha was 0.89.

#### 2.2.3 Anxiety

To gauge anxiety severity among adolescents, the investigation utilized the Chinese adaptation of the Depression Anxiety Stress Scales-21 [DASS-21; Lovibond et al. (45); adapted by Gong et al. (46)], specifically administering the anxiety subscale. This instrument comprises seven items scored on a 4-point Likert metric (1 = “strongly disagree”, 4 = “strongly agree”). The cumulative score reflected overall anxiety level. Increased scores denoted greater anxiety severity. In this study, the sample's Cronbach's alpha was 0.86.

#### 2.2.4 Cognitive flexibility

To evaluate cognitive flexibility among adolescents, the investigation employed the subscale for this construct devised by Chunhui Huang et al. (47) in the executive function scale (e.g., “When plans get changed, I feel completely thrown off balance emotionally.”). The scale consists of eight items (1 = “often”, 3 = “never”). The summed score across all eight items represented the overall cognitive flexibility level. Elevated scores reflected high levels of cognitive flexibility among participants. In this study, the sample's Cronbach's alpha was 0.84.

### 2.3 Statistical analysis

Data analysis was conducted using SPSS 26.0 and R/lavaan. Harman's single-factor test was first used to evaluate common method bias, with a first-factor variance below 40% interpreted as indicating no dominant single factor (48). Given the cross-sectional self-report design, this test was treated as a preliminary screening procedure rather than definitive evidence that common method variance was absent. Therefore, we further conducted a CFA-based unmeasured latent method construct (ULMC) test using item-level data. A baseline four-factor measurement model was specified for physical activity, cognitive flexibility, anxiety, and short-form video addiction. A second model added an orthogonal common latent method factor loading equally on all observed items. Fit indices between the baseline CFA model and the ULMC model were compared to evaluate whether adding a common method factor substantially improved model fit. Descriptive statistics and bivariate correlations were then calculated for demographic characteristics and core study variables. Prior to analysis, the distributions of the study variables were examined. Skewness and kurtosis values were within acceptable ranges (|skewness| < 2, |kurtosis| < 7), supporting the suitability of the data for subsequent parametric analyses. Therefore, no data transformation was performed. The hypothesized chain mediation model was tested using the PROCESS macro (Model 6) with 5,000 bootstrap resamples. Gender, age, and only-child status were included as covariates. Parental education, household income, and academic performance were not available in the dataset and therefore could not be included as additional covariates. Because all variables were measured at the same time point, the mediation model was used to estimate indirect statistical associations rather than temporal or causal effects.

## 3 Result

### 3.1 Common method deviation test

To examine common method bias, Harman's single-factor test was conducted. The first unrotated factor explained 38.79% of the total variance, which was below the 40% threshold, and the unrotated factor analysis yielded two factors with eigenvalues greater than one. These results suggest that no single factor dominated the covariance structure. To provide a more rigorous supplementary assessment, a CFA-based ULMC test was further performed. The baseline four-factor CFA model yielded chi-square = 1,596.236, df = 428, CFI = 0.803, TLI = 0.785, RMSEA = 0.074, and SRMR = 0.077. After adding the common latent method factor, the ULMC model yielded chi-square = 1,538.996, df = 427, CFI = 0.812, TLI = 0.795, RMSEA = 0.073, and SRMR = 0.084. The improvement in fit was small (Delta CFI = 0.010, Delta TLI = 0.010, Delta RMSEA = −0.002). Thus, although residual common method variance cannot be completely ruled out, the supplementary ULMC results did not indicate a large method effect sufficient to overturn the substantive interpretation of the observed associations. Given the modest absolute CFA fit indices and the exclusive reliance on self-report measures, these analyses should not be interpreted as fully ruling out common method bias.

### 3.2 Correlation analysis between variables

Table 1 reveals that physical activity demonstrated a positive relationship with cognitive flexibility among adolescents (*r* = 0.240, *p* < 0.001), whereas it exhibited negative associations with both short-form video (*r* = −0.274, *p* < 0.001) and anxiety (*r* = −0.252, *p* < 0.001). Short-form video addiction displayed a marked negative relationship with cognitive flexibility (*r* = −0.462, *p* < 0.001) and a significant positive association with anxiety (*r* = 0.502, *p* < 0.001). Cognitive flexibility displayed a significant negative correlation with adolescent anxiety (*r* = −0.493, *p* < 0.001).

**Table 1:** Correlation analysis.

Correlation analysis between variables	1	2	3	4	5	6
1 Gender	–					
2 Age	−0.051	–				
3 Physical activity	−0.360^***^	0.026	–			
4 Short-form video addiction	0.255^***^	0.042	−0.274^***^	–		
5 Cognitive flexibility	0.210^***^	−0.040	0.240^***^	−0.462^***^	–	
6 Anxiety	0.195^***^	−0.055	−0.252^***^	0.502^***^	−0.493^***^	–
M/Proportion	–	15.36	23.38	37.44	17.77	13.82
SD	–	0.54	27.14	10.32	3.56	4.71
|Skewness|	–	0.721	1.471	0.039	0.215	0.554
|Kurtosis|	–	0.093	1.240	0.081	0.294	0.058

Gender was dummy-coded for correlation analysis; no mean or SD is reported for gender. ^***^p < 0.001.

### 3.3 Mediation model checking

After including gender, age, and only-child status as covariates, physical activity was significantly and negatively associated with short-form video addiction (beta = −0.208, SE = 0.046, *p* < 0.001) (Table 2). In the mediation model, the residual association between physical activity and short-form video addiction remained significant (beta = −0.089, SE = 0.041, *p* < 0.05). Physical activity was positively associated with cognitive flexibility (beta = 0.192, SE = 0.047, *p* < 0.001) and negatively associated with anxiety (beta = −0.123, SE = 0.042, *p* < 0.01). Cognitive flexibility was negatively associated with both anxiety (beta = −0.450, SE = 0.040, *p* < 0.001) and short-form video addiction (beta = −0.255, SE = 0.043, *p* < 0.001), whereas anxiety was positively associated with short-form video addiction (beta = 0.337, SE = 0.043, *p* < 0.001).

**Table 2:** Chain mediation model test.

Outcome variable	Predicted variable	β	SE	*t*	*R* ^2^	*F*
Short-form video addiction	Physical activity	−0.208	0.046	−4.530^***^	0.108	14.657^***^
	Age	0.055	0.043	1.271		
	Gender	0.185	0.046	4.013^***^		
	Only-child status	0.018	0.043	0.417		
Cognitive flexibility	Physical activity	0.192	0.047	4.102^***^	0.073	9.640^***^
	Age	−0.029	0.044	0.654		
	Gender	0.129	0.047	2.748^**^		
	Only-child status	0.048	0.044	0.110		
Anxiety	Physical activity	−0.123	0.042	−2.891^**^	0.267	35.432^***^
	Cognitive flexibility	−0.450	0.040	−11.160^***^		
	Age	−0.035	0.039	−0.901		
	Gender	0.056	0.042	1.321		
	Only-child status	0.035	0.039	0.905		
Short-form video addiction	Physical activity	−0.089	0.041	−2.192^*^	0.344	42.337^***^
	Cognitive flexibility	−0.255	0.043	−5.959^***^		
	Anxiety	0.337	0.043	7.832^***^		
	Age	0.078	0.037	2.112^*^		
	Gender	0.114	0.040	2.854^**^		
	Only-child status	0.004	0.037	0.111		

^*^p < 0.05; ^**^p < 0.01; ^***^p < 0.001.

**Figure 2:**
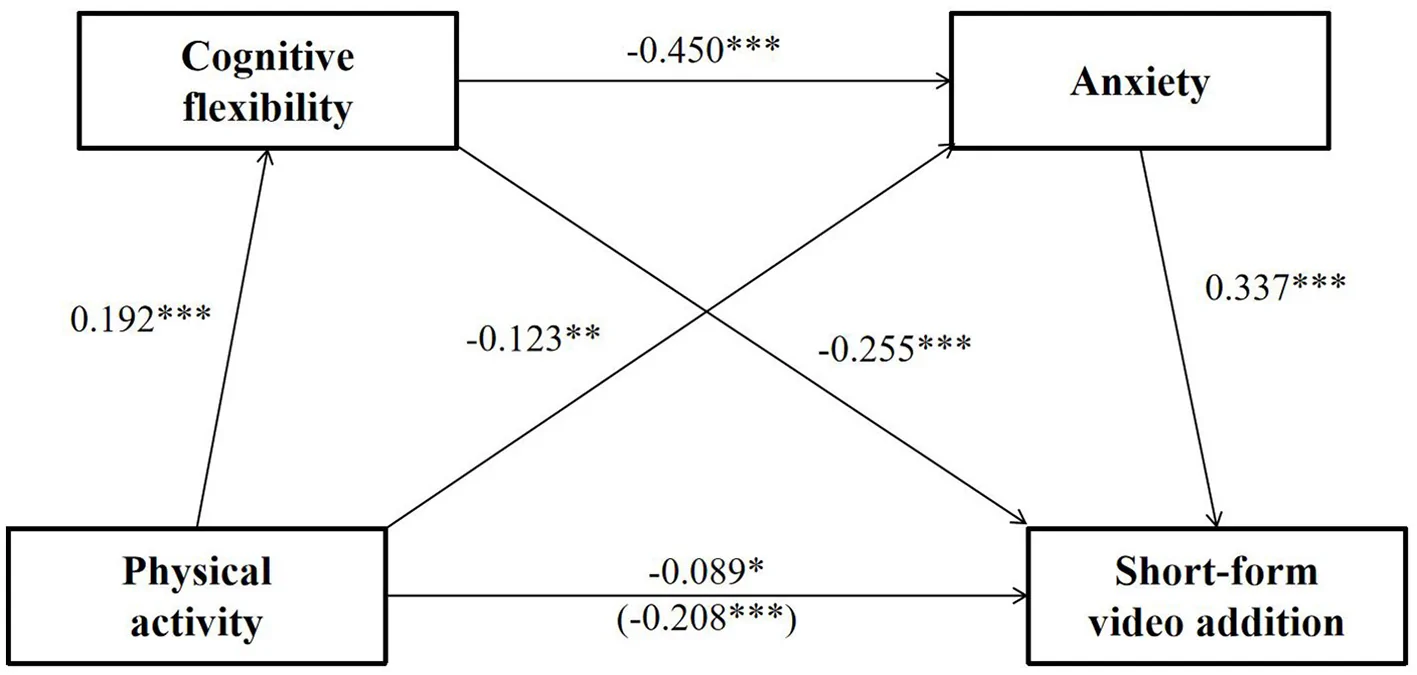
Chain mediation model. ^*^p < 0.05; ^**^p < 0.01; ^***^p < 0.001.

As shown in Table 3, the bootstrapped indirect associations supported the statistical mediating roles of cognitive flexibility and anxiety. The indirect association through cognitive flexibility alone was significant (effect size = −0.049, SE = 0.018, 95% CI [−0.088, −0.019]), accounting for 23.56% of the total association. The indirect association through anxiety alone was also significant (effect size = −0.041, SE = 0.018, 95% CI [−0.079, −0.010]), explaining 19.71% of the total association. The serial indirect association from physical activity to cognitive flexibility to anxiety to short-form video addiction was significant (effect size = −0.029, SE = 0.010, 95% CI [−0.050, −0.013]), accounting for 13.94% of the total association. The total indirect association (effect size = −0.120, SE = 0.026, 95% CI [−0.175, −0.073]) accounted for 57.69% of the association between physical activity and short-form video addiction, with all confidence intervals excluding zero. These estimates describe statistical pathways within the hypothesized model and should not be interpreted as evidence of causal sequence. A comprehensive summary of all indirect effect estimates appears in Table 3, and the hypothesized structural configuration is illustrated in Figure 2.

**Table 3:** Path analysis of the chain mediation model.

Path of mediation	Effect size	SE	Bootstrap 95% CI	Percentage of mediated effects
Total effect	−0.208	0.046	−0.299, −0.118	57.69%
Direct effect	−0.089	0.041	−0.169, −0.009	
Total indirect effect	−0.120	0.026	−0.175, −0.073	
Physical activity -> Cognitive flexibility -> Short-form video addiction	−0.049	0.018	−0.088, −0.019	23.56%
Physical activity -> Anxiety -> Short-form video addiction	−0.041	0.018	−0.079, −0.010	19.71%
Physical activity -> Cognitive flexibility -> Anxiety -> Short-form video addiction	−0.029	0.010	−0.050, −0.013	13.94%

## 4 Discussion

### 4.1 Interpretation of key findings

This study examined the association between physical activity and short-form video addiction among adolescents and tested the chain mediating roles of cognitive flexibility and anxiety. The results showed that higher physical activity was associated with lower short-form video addiction, supporting the proposed hypothesis. This finding is consistent with previous studies reporting an inverse association between physical activity and problematic digital-media use (33, 42). One possible explanation is that physical activity enhances self-regulation, strengthens emotion regulation, and promotes adaptive coping resources, thereby reducing the likelihood of excessive engagement with short-form video platforms (49).

The negative association between physical activity and short-form video addiction may be explained by both behavioral substitution and psychological-resource mechanisms. From a behavioral perspective, greater engagement in physical activity may reduce the amount of discretionary time available for short-form video use (49). From a psychological perspective, regular physical activity has been shown to strengthen self-control, improve mood regulation, and promote more adaptive coping and daily routines (50). These mechanisms are particularly relevant during adolescence, a developmental period characterized by heightened reward sensitivity and ongoing maturation of executive functions. In addition, physical activity may provide structured offline routines, peer interaction, and alternative sources of reward and mastery. These experiences may reduce adolescents' reliance on algorithm-driven digital rewards and may help explain why higher physical activity was associated with lower short-form video addiction in the present sample.

The mediation results further indicated that cognitive flexibility and anxiety partly explained the association between physical activity and short-form video addiction. Physical activity was positively associated with cognitive flexibility and negatively associated with anxiety. In turn, higher cognitive flexibility and lower anxiety were associated with lower short-form video addiction (34, 51). These findings suggest that physical activity may not only directly reduce problematic short-form video use but may also operate through a cognitive-emotional pathway. The serial pathway was specified because cognitive flexibility may help adolescents reinterpret stressors and regulate negative affect before anxiety escalates. However, this ordering is not definitive. Anxiety may also impair cognitive flexibility by narrowing attentional scope and promoting rigid threat-related thinking, and problematic short-form video use may further reinforce anxiety and cognitive rigidity. Future longitudinal studies should compare these competing and potentially reciprocal models.

The total indirect effect accounted for 57.69% of the total association between physical activity and short-form video addiction. Specifically, cognitive flexibility, anxiety, and the serial pathway from cognitive flexibility to anxiety all contributed to the indirect effect. This indicates that more than half of the protective association of physical activity was statistically explained by these cognitive and emotional mechanisms (52–54). However, because the data were cross-sectional, the results should be interpreted as evidence of associations rather than causal effects. Alternative explanations, including the possibility that excessive short-form video use reduces time available for physical activity, cannot be ruled out. Therefore, the present model should be understood as a theoretically informed statistical model rather than a confirmed developmental sequence.

### 4.2 Implications for practice

The findings suggest that school- and family-based prevention programs for short-form video addiction may benefit from integrating regular physical activity with cognitive flexibility training and anxiety-management strategies. Rather than relying only on restrictive rules or direct confrontation, educators and parents may help adolescents build structured routines, increase opportunities for enjoyable physical activity, and develop adaptive coping strategies for negative emotions. Such multidimensional interventions may be more acceptable and developmentally appropriate for adolescents. Because no intervention was conducted, these implications should be regarded as preliminary and hypothesis-generating rather than as evidence that physical-activity programs will necessarily reduce short-form video addiction.

### 4.3 Limitations and future directions

Several limitations should be noted. First, although the final sample size was adequate for the planned mediation analysis, the study used a convenience sample from three schools in one province; therefore, the representativeness of the findings is limited, especially with respect to urban-rural differences and socioeconomic diversity. Second, all measures were based on adolescent self-report at a single time point, which may introduce recall bias, social desirability bias, and common method variance. Future studies should incorporate objective physical activity data (e.g., accelerometers, pedometers, or school exercise records) and parent- or teacher-reported indicators of digital-media problems. Third, the physical activity scale showed marginal internal consistency (Cronbach's alpha = 0.67), and the multiplicative scoring method may increase distributional skewness; future research should use more reliable and objective physical activity measures. Fourth, parental education, household income, academic performance, and other potential confounders were not available in the present dataset. Finally, the cross-sectional design prevents causal inference. Longitudinal and intervention studies are needed to determine whether increasing physical activity can reduce short-form video addiction through changes in cognitive flexibility and anxiety. In addition, unmeasured confounders such as socioeconomic status, parental education, academic stress, daily screen time, sleep duration, parental monitoring, family media rules, depressive symptoms, and other psychosocial characteristics may partially account for the observed associations. Future studies should include these variables and compare competing mediation directions to clarify whether the associations are unidirectional, reverse, or reciprocal.

## 5 Conclusion

This study identified three indirect pathways linking physical activity to adolescent short-form video addiction: a pathway through cognitive flexibility, a pathway through anxiety, and a serial pathway through cognitive flexibility and anxiety. Higher physical activity was associated with lower short-form video addiction, and this association was partly explained by higher cognitive flexibility and lower anxiety. These findings provide preliminary evidence that physical activity may be a useful component of multidimensional strategies to prevent problematic short-form video use among adolescents. Overall, the findings provide preliminary associational evidence and should be followed by longitudinal, experimental, and multi-informant studies before causal or intervention conclusions are drawn.

## Data Availability

The raw data supporting the conclusions of this article will be made available by the authors, without undue reservation.
